# The Midwives’ Data Hub as Workforce Policy Intelligence for Staffing, Well‐Being and Equity

**DOI:** 10.1111/inr.70240

**Published:** 2026-09-15

**Authors:** Kazumi Kubota, Yoko Shimpuku

**Affiliations:** ^1^ Department of Women's Health Population Sciences National Center for Child Health and Development Setagaya Japan; ^2^ Department of Healthcare Information Management The University of Tokyo Hospital Tokyo Japan; ^3^ Shimonoseki City University Yamaguchi Japan; ^4^ Graduate School of Biomedical and Health Sciences Hiroshima University Hiroshima Japan

**Keywords:** digital workforce data, equity, health workforce, midwifery, nursing policy, staffing, workforce well‐being

## Abstract

**Aim:**

To examine how the Midwives’ Data Hub can function as workforce policy intelligence to support staffing, workforce well‐being and equity.

**Background:**

Midwifery shortages, maldistribution and unsustainable workloads threaten equitable maternity care. Policymakers need midwifery‐specific information that connects workforce indicators with education, deployment, retention, regulation, service design and financing.

**Policy Context:**

Global workforce monitoring has improved visibility of health workforce challenges, but aggregate indicators may obscure midwifery‐specific risks. In settings such as Japan, demographic change, regional service variation and workforce constraints create a need for context‐sensitive planning.

**Description of Innovation:**

The Midwives’ Data Hub is framed as an enabling layer of workforce policy intelligence, not as a direct staffing intervention. It can organise indicators across workforce distribution, education, regulation, scope of practice, metadata and service context to support policy deliberation.

**Key Outcomes:**

The analysis proposes a policy logic in which data visibility supports problem definition, stakeholder interpretation, selection of policy packages, implementation monitoring and policy revision. Potential policy levers include education planning, deployment support, retention measures, scope‐of‐practice optimisation, work design and financing. Governance risks include incomplete data, weak comparability, punitive dashboard use and insufficient professional participation.

**Conclusion:**

Hub‐informed planning may support better workforce decisions only when linked to resources, leadership, professional judgement and accountable implementation.

**Implications for Nursing:**

Workforce data should strengthen dialogue about workload, supervision, rostering, continuing professional development and midwives’ professional voice, rather than replace local judgement.

**Implications for Health Policy:**

Policymakers should embed the Midwives’ Data Hub in participatory governance that connects indicators to financing, education, retention, service equity, workforce equity and policy revision. This approach treats workforce intelligence as a basis for accountable action rather than simple performance comparison.

## 1 Background

Midwives are central to equitable maternity care. International policy documents identify midwifery as a workforce priority for universal health coverage, quality of care and health equity. The WHO Global Strategic Directions for Nursing and Midwifery 2021–2025 emphasise education, jobs, leadership and service delivery as interconnected policy areas for strengthening nursing and midwifery contributions to population health (World Health Organization 2021a). The State of the World's Midwifery 2021 highlighted substantial shortages and maldistribution of midwives and the consequences of underinvestment (UNFPA et al. 2021). Evidence from the Lancet Series on Midwifery further shows that midwifery contributes to quality maternal and newborn care when supported by education, regulation, effective deployment and enabling work environments (Renfrew et al. 2014; ten Hoope‐Bender et al. 2014). Evidence on midwife‐led continuity models also supports the value of midwifery‐led care for women and newborns (Sandall et al. 2016). Expanding access to midwife‐delivered interventions has also been estimated to contribute substantially to reductions in maternal and neonatal mortality and stillbirths (Nove et al. 2021).

Workforce shortages are not experienced only as numerical gaps. They are translated into uneven workloads, service consolidation, limited supervision, reduced access in underserved areas and increased physical and emotional strain. Evidence from nursing staffing research shows that staffing levels, skill mix and missed care are associated with patient outcomes and safety, although these relationships are mediated by organisational context and implementation arrangements (Aiken et al. 2014; Ball et al. 2018; Griffiths et al. 2019; McHugh et al. 2021). The Job Demands–Resources model suggests that work‐related strain emerges when demands such as workload, time pressure and emotional burden exceed resources such as staffing, autonomy, supervision, leadership and organisational support (Bakker and Demerouti 2017). From this perspective, digital workforce data cannot by themselves improve well‐being. Rather, it may support better policy decisions when it helps decision‐makers identify where demands and resources are misaligned and where staffing, supervision, retention or work‐design responses are needed (Dall'Ora et al. 2020; Sidhu et al. 2020).

Global workforce data initiatives have improved the visibility of health workforce challenges. The National Health Workforce Accounts provide a standardised approach to monitoring workforce stock, distribution, education, migration, expenditure and governance (World Health Organization 2023). However, policymakers often require more granular and policy‐ready information to connect workforce data with decisions about education capacity, deployment, retention, scope of practice, service configuration and financing. Recent work on staffing systems emphasises that workforce tools are most useful when they augment professional judgement, account for local context and are linked to governance and accountability arrangements (Allen et al. 2025).

The Midwives’ Data Hub is positioned in this paper as a complementary form of workforce policy intelligence. Its potential contribution lies not simply in collecting more data, but in organising midwifery‐specific indicators in ways that make workforce risks easier to identify, interpret and discuss (International Confederation of Midwives 2025). This allows decision‐makers to ask more actionable questions: where services are vulnerable, which workforce groups are carrying disproportionate burden, which policy levers are available and how changes should be monitored over time.

Japan provides a useful illustrative context because rapid population ageing, low fertility, regional variation in service access and pressure to sustain maternity services intersect with workforce constraints (OECD 2024; Cabinet Office 2025). Declining birth numbers may lead to service consolidation, but low birth volume does not necessarily mean low workforce need, especially where travel time, emergency readiness, on‐call burden and professional isolation remain important.

### 1.1 Aim

To examine how the Midwives’ Data Hub can function as workforce policy intelligence to support staffing, workforce well‐being and equity.

This paper makes three contributions. First, it reframes the Midwives’ Data Hub as workforce policy intelligence rather than as a stand‐alone digital intervention. Second, it clarifies a policy pathway linking midwifery‐specific indicators to problem definition, stakeholder deliberation, policy package selection, monitoring and revision. Third, it distinguishes service‐level equity from workforce‐level equity, arguing that equitable maternity access should not be achieved by intensifying pressure on an already stretched midwifery workforce.

In this paper, service‐level equity refers to timely and appropriate access to maternity care for women, newborns and families, whereas workforce‐level equity refers to fair workloads, supervision, career development and protection from unsustainable work intensity among midwives.

### 1.2 Sources of Evidence and Analytic Approach

This paper is a structured, non‐empirical policy analysis, not a systematic, scoping or narrative review. It does not report primary data collection, participant recruitment or outcome evaluation. Its purpose is to develop a policy logic for how the Midwives’ Data Hub may function as workforce policy intelligence to support decision‐making on staffing, workforce well‐being and equity.

The analysis draws on three categories of evidence: international policy documents on the health and midwifery workforce; peer‐reviewed literature on workforce planning, staffing, well‐being, data governance and policy implementation; and publicly available information on the Midwives’ Data Hub and the Japanese maternity care context (International Confederation of Midwives 2025; OECD 2024; Cabinet Office 2025). Sources were selected purposively rather than through a systematic review. Priority was given to documents and studies directly relevant to midwifery workforce distribution, staffing, workload, workforce well‐being, health workforce data systems, policy implementation, equity and governance.

The synthesis was conducted narratively and conceptually. Existing workforce data initiatives were reviewed to identify the policy gap addressed by the Midwives’ Data Hub. Relevant literature was then used to clarify plausible pathways linking workforce data visibility to policy action, staffing decisions and workforce well‐being. Japan was used as a worked example to illustrate how hub indicators could inform prefectural workforce planning.

The analytic approach was informed by three complementary lenses. The policy cycle was used to organise the pathway from problem identification to policy formulation, implementation, monitoring and revision. The health policy triangle was used to consider how content, context, actors and process shape whether workforce data are translated into action (Walt and Gilson 1994). The Job Demands–Resources model was used to explain how staffing policies may influence well‐being through workload, autonomy, supervision, support and organisational resources (Bakker and Demerouti 2017). Complex intervention guidance informed the treatment of the Hub as a programme theory requiring contextual adaptation, stakeholder engagement and empirical evaluation (Skivington et al. 2021).

## 2 Discussion

Several limitations should be acknowledged at the outset. This is a non‐empirical policy analysis and does not provide causal evidence that the Hub improves staffing, retention or well‐being. The Japan example is illustrative rather than generalisable. The value of the Hub therefore lies in the policy logic it offers, which requires empirical evaluation in specific governance and workforce contexts.

### 2.1 The Midwives’ Data Hub as Workforce Policy Intelligence

This paper positions the Midwives’ Data Hub as workforce policy intelligence rather than as a direct staffing intervention. Digital workforce platforms can make information easier to locate, compare and interpret, but they do not by themselves create posts, redistribute workload, improve retention or reduce burnout. Their policy value depends on whether data are connected to decision‐making authority, financing, professional judgement, implementation capacity and accountability for action (Walt and Gilson 1994; World Health Organization 2023; Allen et al. 2025).

The Hub's contribution lies in organising midwifery‐specific workforce information in a way that supports policy deliberation across the workforce pathway. Midwifery workforce problems may reflect insufficient education capacity, weak retention, restrictive deployment arrangements, limited scope of practice, unattractive working conditions, geographic isolation or service reconfiguration. A policy‐ready data hub can help clarify which problem is most relevant in a given context and which policy response is most appropriate.

### 2.2 Added Value Beyond Existing Workforce Data Initiatives

Several international workforce data initiatives already provide essential information for policy and planning. The National Health Workforce Accounts offer a standardised approach to monitoring health workforce stock, distribution, education, migration, expenditure and governance (World Health Organization 2023). The State of the World's Midwifery has drawn global attention to shortages, maldistribution and underinvestment in midwifery (UNFPA et al. 2021). The Hub is complementary to these initiatives because it focuses specifically on midwifery and can support interpretation of workforce risks across education, regulation, deployment, service delivery and contextual conditions (International Confederation of Midwives 2025).

The added value of the Midwives’ Data Hub is different and complementary. First, it is midwifery‐specific. Aggregate nursing or health workforce indicators may obscure the distinctive role, education pathway, regulatory position and service contribution of midwives. Second, it can connect multiple domains of workforce policy, including education, regulation, distribution, service delivery, professional role and contextual metadata. Third, it can support interpretation by making visible not only workforce numbers, but also the conditions under which those numbers are meaningful.

The Hub should therefore not be framed as replacing existing global monitoring systems. Its contribution is to translate broad workforce concerns into more actionable policy questions for midwifery.

### 2.3 From Data Visibility to Policy Action and Workforce Well‐Being

The pathway from workforce data to well‐being is indirect. Workforce data do not reduce workload or burnout on their own. However, they may support policy decisions that change the balance between job demands and organisational resources. From a Job Demands–Resources perspective, the Hub may contribute to well‐being only when its indicators are used to identify imbalances and guide corrective action that reduces excessive demands or strengthens resources such as staffing, supervision, autonomy and organisational support (Bakker and Demerouti 2017; Dall'Ora et al. 2020; Sidhu et al. 2020).

This pathway can be understood as a sequence of policy steps: problem definition, stakeholder interpretation, selection of a policy package, implementation monitoring and policy revision. Better workforce data are necessary for more transparent and accountable planning, but they are not sufficient for staffing sustainability or workforce well‐being. Improvements depend on whether decision‐makers act on the information, whether resources are available and whether midwives and service users are involved in interpreting priorities and evaluating consequences.

**FIGURE 1:**
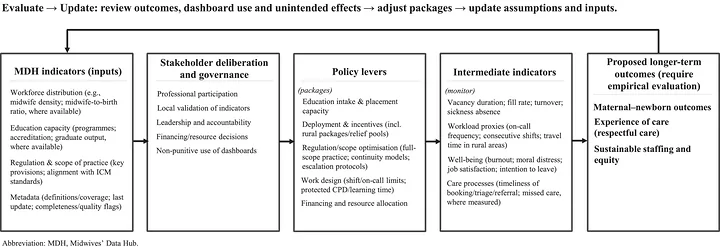
Programme theory of the Midwives’ Data Hub as workforce policy intelligence. The figure presents a programme theory of the Midwives’ Data Hub as an enabling layer of workforce policy intelligence rather than as a direct staffing intervention. Midwifery‐specific indicators inform problem definition, stakeholder deliberation and local validation. Policy actors then select context‐sensitive levers, including education, deployment, retention, regulation, service redesign, work design, financing and resource allocation. Implementation is monitored through service‐level and workforce‐level indicators, with feedback used to revise policy packages. The proposed longer‐term outcomes, including staffing sustainability, workforce well‐being and equitable service access, require empirical evaluation and depend on resources, leadership, financing, professional participation, transparent governance and accountable, non‐punitive use of workforce data. The model does not assume direct causal effects from data to outcomes; rather, it identifies governance conditions under which workforce intelligence may support accountable policy action. MDH, Midwives’ Data Hub.

Figure 1 presents the programme theory proposed in this paper. It depicts the Midwives’ Data Hub as workforce policy intelligence that links midwifery‐specific indicators to policy deliberation, context‐sensitive policy levers, implementation monitoring and feedback. The model does not assume that data visibility directly improves staffing or well‐being; rather, it identifies the conditions under which data may support more accountable policy action, including resources, leadership, professional participation and governance.

### 2.4 Worked Example: Hub‐Informed Prefectural Workforce Planning in Japan

Japan provides a useful worked example because rapid population ageing, low fertility, regional variation in service access and pressure to sustain maternity services intersect with workforce constraints (OECD 2024; Cabinet Office 2025). Declining birth numbers may lead to consolidation of maternity services, but lower birth volume does not necessarily mean lower workforce need. In geographically dispersed areas, safe and equitable maternity care may still require emergency readiness, travel‐time consideration, continuity of care, supervision capacity and sustainable on‐call arrangements.

A Midwives’ Data Hub could support prefectural workforce planning through five linked steps. First, policymakers could develop a prefectural workforce profile by combining midwifery‐specific indicators with contextual information on births, maternity facilities, travel time, education capacity, vacancies, age distribution, turnover, on‐call burden and service configuration. The aim would not be to rank prefectures, but to identify areas where multiple risks converge. In practical terms, the Hub would be most useful when indicators are interpreted as a risk profile rather than as isolated measures. A policy review could be triggered when several risks converge, such as persistent vacancies, long travel times, high on‐call burden, limited supervision capacity and declining early‐career retention.

Second, the profile could be used to classify the dominant workforce problem. Some prefectures may face a numerical supply problem; others may face maldistribution, retention, work‐design or education‐pipeline problems. Differentiating these problem types is essential because each requires a different policy response.

Third, policymakers could select a context‐sensitive policy package. Short‐term responses may include deployment support, protected rest after on‐call work, transport or housing support and strengthened supervision. Medium‐term responses may include rural retention incentives, transition‐to‐practice programmes and continuing education. Longer‐term responses may include education‐capacity planning, service redesign, scope‐of‐practice review and financing reform. Evidence on rural and remote workforce retention suggests that such responses are most effective when they combine professional, organisational, financial and personal support rather than relying on single incentives (Russell et al. 2021; World Health Organization 2021b).

Fourth, implementation should be monitored using both service and workforce indicators, including service access, referral patterns, vacancy duration, turnover, overtime, on‐call frequency, supervision, job satisfaction and intention to leave. Fifth, monitoring results should feed back into policy revision. The Japanese example is not presented as a model for direct transfer, but as an adaptable policy logic for contexts where national workforce totals conceal regional maldistribution, service‐access inequities or unsustainable work patterns.

### 2.5 Political Economy and Governance Risks of Workforce Data

The use of workforce data is not politically neutral. Indicators can make shortages, maldistribution and workload pressures more visible, but visibility alone does not guarantee policy action. Decisions about staffing, education capacity, service configuration, regulation and financing are shaped by budgets, institutional priorities, professional boundaries, labour‐market conditions and political negotiation. The Hub should therefore be understood not only as a technical platform but also as part of a broader policy process in which evidence, interests, power and implementation capacity interact (Walt and Gilson 1994). Workforce data governance should also be aligned with rights‐based approaches to protecting health and care workers, ensuring decent work and avoiding discriminatory or punitive use of workforce indicators (Friedman et al. 2023).

There is a risk that workforce indicators are used for punitive comparison rather than supportive improvement. Simplistic comparisons may misclassify low‐volume rural services as inefficient or overlook hidden workload in apparently well‐staffed urban services. Data quality and comparability are further governance concerns. Workforce data may be incomplete, inconsistently defined or difficult to compare across regions, facility types and employment arrangements. For the Hub to support fair policy decisions, indicators should be accompanied by transparent definitions, metadata, caveats and mechanisms for local validation (World Health Organization 2023). Where advanced analytic functions are incorporated, transparency, explainability, human oversight and contestability are also essential to avoid bias, surveillance and inappropriate automation of workforce decisions (World Health Organization 2021c; Park 2025).

The ethical purpose of workforce data should be to strengthen accountability for support, not only accountability for performance. Data that expose workforce pressure without enabling support may increase surveillance and blame; data linked to financing, professional voice and policy revision may support safer and fairer workforce planning.

### 2.6 From Indicators to Policy Judgement

Workforce indicators should not be interpreted as automatic decision rules. The value of the Midwives’ Data Hub lies in making workforce risks more visible and discussable, but staffing decisions still require professional judgement, contextual interpretation and political commitment. Safe staffing is not determined by headcount alone. It depends on workload, acuity, service configuration, scope of practice, supervision capacity, geography, transport time, educational pipelines and organisational support.

Recent work on staffing optimisation highlights that formal staffing systems are most useful when they augment, rather than replace, professional judgement and when they are adapted to local service conditions (Allen et al. 2025). For the Midwives’ Data Hub, this implies that indicators should be linked to explicit policy questions: which services are at risk, which groups of workers are carrying the greatest burden, which policy lever is most appropriate and what unintended consequences might follow.

If more advanced analytic functions are incorporated into workforce platforms in the future, they should remain explainable, auditable and contestable. Midwives, managers, service users and policymakers should be able to understand how indicators are defined, what data are missing and how conclusions are reached. The Hub should support shared judgement, transparency and accountability for remedial action, not replace professional expertise or democratic policy deliberation (World Health Organization 2021c; Park 2025).

### 2.7 Future Research Agenda

Future research should evaluate the Hub as part of a policy process rather than as a stand‐alone digital intervention. Research should examine whether, how, for whom and under what conditions Hub‐informed policy processes influence staffing sustainability, workforce well‐being and equity, consistent with guidance for evaluating complex interventions (Skivington et al. 2021).

Natural experiments, comparative interrupted time‐series designs and difference‐in‐differences approaches may help assess whether Hub‐informed planning is associated with changes in service access, vacancy duration, turnover, overtime, on‐call burden or intention to leave (Craig et al. 2012). Realist evaluation would also be valuable for examining when dashboards lead to supportive investment, when they are ignored and when they create unintended pressure or punitive accountability (Pawson and Tilley 1997). Such evaluation should examine both service‐level equity and workforce‐level equity.

### 2.8 Implications for Nursing Practice

For nursing and midwifery practice, the Midwives’ Data Hub may help make workforce pressures more visible and discussable. Headcount alone cannot capture on‐call burden, overtime, travel time, professional isolation, limited supervision or emotional demands. By linking workforce indicators with contextual information, the Hub may help managers and professional leaders identify where apparent staffing adequacy masks unsafe or unsustainable work patterns, including missed care, excessive workload, burnout risk or retention risk (Ball et al. 2018; Bloxsome et al. 2019; Harvie et al. 2019; Matthews et al. 2024).

The Hub should also strengthen professional voice. Midwives should be involved in interpreting indicators because they can identify hidden workload, moral distress and service risks that may not be visible in administrative data. Used appropriately, workforce data can support dialogue about rostering, supervision, continuing professional development and targeted support. The practical implication is that dashboards should trigger local questions and action, not replace professional judgement, relational leadership or professional voice.

### 2.9 Implications for Nursing Policy

For nursing policy, the Hub should be embedded in accountable workforce governance rather than treated as a stand‐alone technological solution. Its value depends on whether indicators are linked to financing, education planning, deployment, retention, regulation, service configuration and policy revision. This aligns with international priorities on education, jobs, leadership and service delivery for nursing and midwifery (World Health Organization 2021a). It also reflects wider policy recognition that workforce well‐being is a systems responsibility requiring sustained organisational and policy action, not only individual resilience (National Academy of Medicine 2022).

Policymakers should use the Hub to monitor both service‐level equity and workforce‐level equity. Service‐level equity concerns access to timely maternity care; workforce‐level equity concerns fair workloads, supervision, career development and protection from unsustainable work intensity. Indicators should be accompanied by transparent definitions, metadata and opportunities for local validation (World Health Organization 2023). Dashboards should not be used as punitive rankings or as tools for cost containment detached from professional and community needs. Their ethical purpose should be to strengthen accountability for support, not only accountability for performance. With appropriate governance, professional participation and investment, the Hub may support fairer and more sustainable midwifery workforce policy.

Three policy messages follow from this analysis. First, workforce data should strengthen, not replace, professional judgement in midwifery staffing decisions. Second, indicators must be linked to policy levers, including staffing standards, education pipelines, retention strategies, work‐environment investment and financing. Third, workforce intelligence should be governed through transparent, participatory and equity‐oriented processes to avoid surveillance, blame or purely cost‐containment uses of data.

### 2.10 Limitations

This paper has several limitations. First, it is a non‐empirical policy analysis and does not provide causal evidence that the Midwives’ Data Hub improves staffing sustainability, workforce well‐being or equity. The proposed pathway should be interpreted as a plausible policy mechanism rather than as evidence of effectiveness. Second, sources were selected purposively rather than through a systematic review. The analysis was designed to develop a policy argument and programme theory, not to provide an exhaustive evidence synthesis.

Third, Japan is used as an illustrative worked example rather than a model for direct transfer to other countries. The proposed prefectural planning logic requires adaptation to local governance arrangements, financing systems, professional regulation, data infrastructure and community needs. Fourth, the value of the Hub depends on the quality, completeness and comparability of the underlying data. Inconsistent definitions, missing data, variation in reporting capacity and limited information on workload, supervision or on‐call burden may produce misleading comparisons. Indicators should therefore be accompanied by transparent definitions, metadata, caveats and opportunities for local validation (World Health Organization 2023).

Finally, the programme theory has not yet been empirically tested. More advanced analytic functions may also create governance risks, including bias, lack of explainability, surveillance and punitive use of indicators. The Hub should therefore remain a tool for shared policy judgement and accountable support, not a substitute for professional expertise or democratic decision‐making (World Health Organization 2021c; Park 2025).

## 3 Conclusion

The Midwives’ Data Hub should be understood as workforce policy intelligence, not as a stand‐alone solution to midwifery staffing shortages or workforce distress. Its value lies in making midwifery‐specific workforce risks more visible, interpretable and actionable for policy deliberation. By connecting indicators on workforce distribution, education, regulation, service need and contextual conditions, the Hub may help policymakers and professional leaders identify where staffing pressure, service‐access risk and workforce well‐being concerns intersect.

However, data visibility is not sufficient to improve staffing sustainability or workforce well‐being. Improvements depend on whether information is linked to financing, leadership, professional participation, supportive work design and accountable implementation. The Japanese worked example illustrates an adaptable policy logic for contexts where national workforce totals conceal regional maldistribution, service‐access inequities or unsustainable work patterns.

Future research should test this policy logic empirically. Natural experiments, realist evaluations and mixed‐methods implementation studies are needed to examine whether, how, for whom and under what conditions Hub‐informed policy processes support staffing decisions, workforce well‐being and equity. Used responsibly, the Midwives’ Data Hub may help shift workforce planning from descriptive monitoring towards more transparent, participatory and accountable policy action.

## Author Contributions

Study concept and design: KK and YS. Identification and review of publicly available sources: KK. Analysis and interpretation: KK and YS. Manuscript writing: KK. Critical revision for important intellectual content: YS. Final approval of the manuscript: KK and YS. Accountability for all aspects of the work: KK and YS.

## Funding

The authors have nothing to report.

## Ethics Statement

This is a non‐empirical policy analysis using publicly available sources and does not involve human participants, personal data collection or intervention.

## Conflicts of Interest

The authors declare no conflicts of interest.

## Use of Generative Artificial Intelligence

Generative artificial intelligence tools were used only for language editing, grammar checking and reference‐formatting support under author supervision. They were not used to generate the study concept, policy analysis, interpretation, conclusions or original intellectual content. The authors reviewed, verified and edited all text and references and take full responsibility for the accuracy, integrity and final content of the manuscript. No confidential, personal or proprietary data were uploaded to generative artificial intelligence tools.

## Data Availability

Data sharing not applicable to this article, as no datasets were generated or analysed during the current study.
